# The role of empagliflozin-induced metabolic changes for cardiac function in patients with type 2 diabetes. A randomized cross-over magnetic resonance imaging study with insulin as comparator

**DOI:** 10.1186/s12933-023-02094-x

**Published:** 2024-01-06

**Authors:** Roopameera Thirumathyam, Erik Arne Richter, Gerrit van Hall, Jens Juul Holst, Mogens Fenger, Jens P. Gøtze, Ulrik Dixen, Niels Vejlstrup, Sten Madsbad, Per Lav Madsen, Nils Bruun Jørgensen

**Affiliations:** 1 Department of Endocrinology and Pulmonary Medicine, Amager and Hvidovre Hospital, Kettegårds Alle 30, 2650 Hvidovre, Denmark; 2 Department of Nutrition, Exercise and Sports, University of Copenhagen, Copenhagen, Denmark; 3 Department of Clinical Biochemistry, Rigshospitalet, Copenhagen, Denmark; 4 Department of Biomedical Sciences, Faculty of Health Sciences, University of Copenhagen, Copenhagen, Denmark; 5 Clinical Metabolomics Core Facility, Rigshospitalet, Copenhagen, Denmark; 6 Department of Clinical Biomedical Sciences, Hvidovre Hospital, Hvidovre, Denmark; 7 Department of Cardiology, Amager and Hvidovre Hospital, Hvidovre, Denmark; 8 Department of Cardiology, Rigshospitalet, Copenhagen, Denmark; 9 Department of Cardiology, Herlev Hospital, Herlev, Denmark; 10 Institute of Clinical Medicine, Copenhagen University, Copenhagen, Denmark

**Keywords:** Sodium-glucose linked transporter 2 inhibition, Cardiac function, Metabolism, Type 2 diabetes, Hyperinsulinemia

## Abstract

**Background:**

Metabolic effects of empagliflozin treatment include lowered glucose and insulin concentrations, elevated free fatty acids and ketone bodies and have been suggested to contribute to the cardiovascular benefits of empagliflozin treatment, possibly through an improved cardiac function. We aimed to evaluate the influence of these metabolic changes on cardiac function in patients with T2D.

**Methods:**

In a randomized cross-over design, the SGLT2 inhibitor empagliflozin (E) was compared with insulin (I) treatment titrated to the same level of glycemic control in 17 patients with type 2 diabetes, BMI of > 28 kg/m^2^, C-peptide > 500 pM. Treatments lasted 5 weeks and were preceded by 3-week washouts (WO). At the end of treatments and washouts, cardiac diastolic function was determined with magnetic resonance imaging from left ventricle early peak-filling rate and left atrial passive emptying fraction (primary and key secondary endpoints); systolic function from left ventricle ejection fraction (secondary endpoint). Coupling between cardiac function and fatty acid concentrations, was studied on a separate day with a second scan after reduction of plasma fatty acids with acipimox. Data are Mean ± standard error. Between treatment difference (ΔT: E–I) and treatments effects (ΔE: E-WO or ΔI: I -WO) were evaluated using Students’ t-test or Wilcoxon signed rank test as appropriate.

**Results:**

Glucose concentrations were similar, fatty acids, ketone bodies and lipid oxidation increased while insulin concentrations decreased on empagliflozin compared with insulin treatment. Cardiac diastolic and systolic function were unchanged by either treatment. Acipimox decreased fatty acids with 35% at all visits, and this led to reduced cardiac diastolic (ΔT: −51 ± 22 ml/s (p < 0.05); ΔE: −33 ± 26 ml/s (ns); ΔI: 37 ± 26 (ns, p < 0.05 vs ΔE)) and systolic function (ΔT: -3 ± 1% (p < 0.05); ΔE: −3 ± 1% (p < 0.05): ΔI: 1 ± 2 (ns, ns vs ΔE)) under chronotropic stress during empagliflozin compared to insulin treatment.

**Conclusions:**

Despite significant metabolic differences, cardiac function did not differ on empagliflozin compared with insulin treatment. Impaired cardiac function during acipimox treatment, could suggest greater cardiac reliance on lipid metabolism for proper function during empagliflozin treatment in patients with type 2 diabetes.

*Trial registration:* EudraCT 2017-002101-35, August 2017.

**Supplementary Information:**

The online version contains supplementary material available at 10.1186/s12933-023-02094-x.

## Background

The EMPA-REG outcome trial was the first to show that the risk of major cardiovascular events was reduced in patients with type 2 diabetes when treated with sodium-glucose co-transporter-2 inhibitors (SGLT2i) [1], but the mechanisms behind this finding are still not fully understood. The cardioprotective effects are present soon after treatment is initiated and have so far been suggested to relate to changes in inflammatory activity [2] or changes in hemodynamics [3] or to a changed overall cardiac metabolism that may benefit cardiac function [4, 5]. In patients with type 2 diabetes, cardiac function and structure are altered independently of myocardial ischemia and hypertension in a disease process collectively known as “diabetic cardiomyopathy” (DCM)[6]. Characterized by left ventricular hypertrophy and impaired diastolic filling, DCM is an early and frequent complication to type 2 diabetes and a risk marker of future cardiovascular events [7]. The severity of DCM has been coupled to the level of whole-body insulin resistance, and an altered cardiac substrate metabolism more dependent on lipid than glucose oxidation as compared to hearts of normal control subjects [8]. In this context, the suggested link between an improved metabolism and cardio-protection induced by SGLT2i treatment is especially interesting, in as much as both systole and diastole are energy requiring processes and hence potentially modifiable [9, 10].

Switching metabolism to improve cardiovascular outcome in type 2 diabetes has been tried before by increasing cardiac glucose utilization with insulin. Since glucose oxidation yields more ATP pr unit oxygen than lipid oxidation, this could in theory pose an advantage during myocardial stress [11]. However, initial promise with glucose-insulin infusions immediately after myocardial infarction, could not be confirmed in later studies [12–14] and an approach with insulin-treatment may even cause early harm after STEMI [14]. Aggressive insulin treatment of hyperglycemia in non-surgical intensive care unit patients is associated with increased mortality, and intensive glycemic control with mostly exogenous insulin, insulin secretagogues, and metformin does not improve and may even worsen cardiovascular risk in individuals with type 2 diabetes [15, 16]. Furthermore, hyperinsulinemia is associated with increased risk of cardiovascular events [17].

SGLT2 inhibition increases renal glucose excretion, lowers plasma glucose and insulin levels and reduces tissue glucose uptake and oxidation. It also increases glucagon release, hepatic glucose production, lipolysis, ketogenesis and lipid oxidation, i.e. metabolic changes opposite to those found with insulin treatment [18, 19]. The potential for such metabolic changes to affect cardiac function is illustrated by the finding that acute lowering of fatty acids (FA) with acipimox causes impaired cardiac contractility in patients with type 2 diabetes and that infusion of ketone bodies to physiological levels improves contractility in patients with heart failure [20, 21]. The metabolic changes with SGLT2i treatment occur early after initiation of therapy to coincide with the cardio-protective effects, and echocardiographic studies have shown that cardiac diastolic function may be improved [22].

The present study aims to evaluate the impact of the metabolic effects of empagliflozin on cardiac function in patients with type 2 diabetes as compared to insulin treatment titrated to the same level of glycemic control in a randomized cross-over design. We hypothesize that the metabolic milieu during treatment with an SGLT2i with reduced insulinemia, higher availability of fatty acids and ketone bodies and greater lipid oxidation would improve cardiac diastolic and systolic function as compared with the lower level of fatty acids and ketone bodies seen during insulin treatment. Secondly, that acute reduction of fatty acids and ketone bodies during treatment with acipimox will impair cardiac function on empagliflozin treatment.

## Methods

### Aim

To evaluate the impact of metabolic changes with empagliflozin treatment on cardiac function during rest and chronotropic stress and compare with insulin treatment titrated to the same level of glycemic control.

### Design and setting

**Fig. 1:**
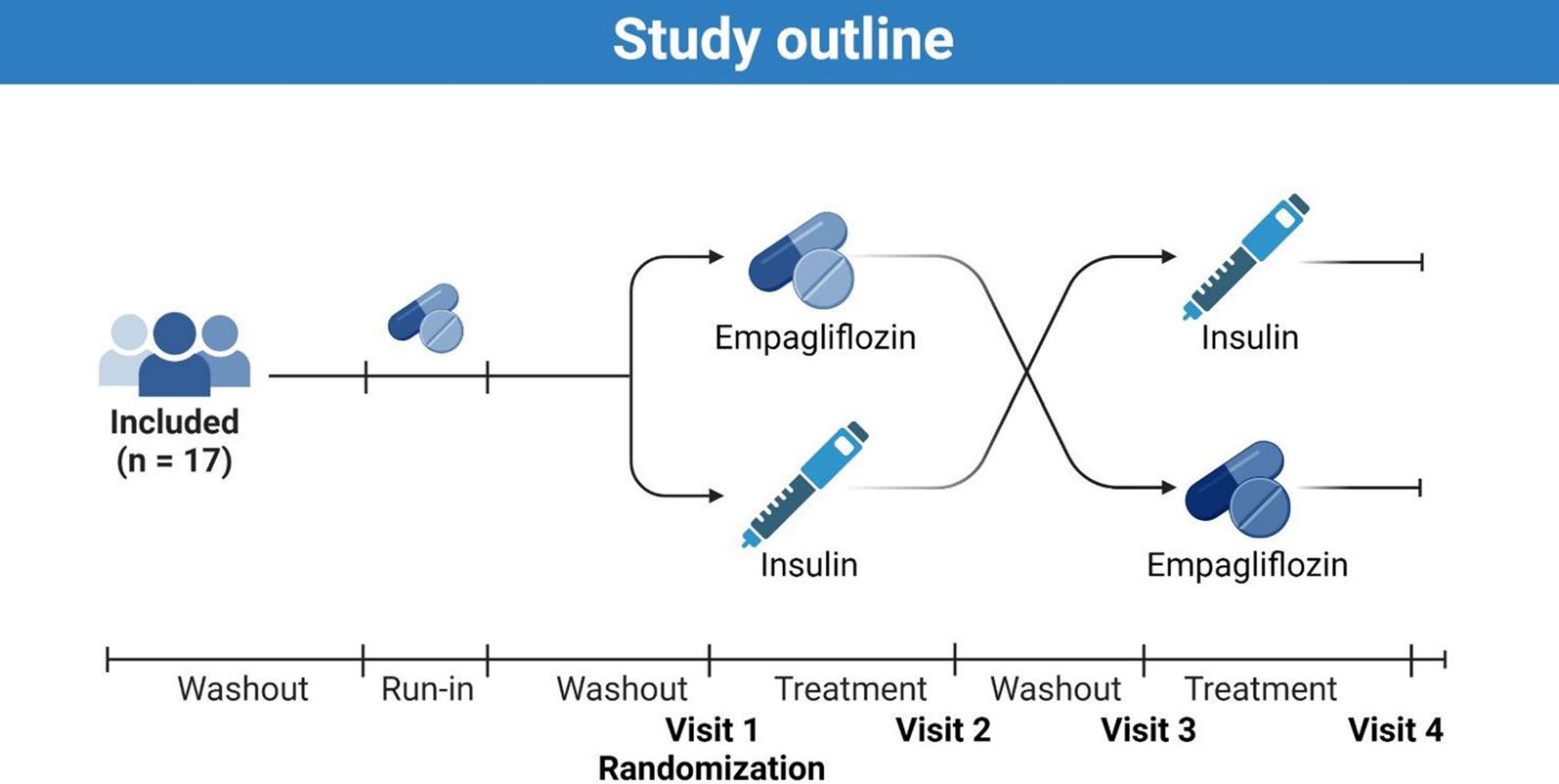
Study outline. Participants were included, to undergo a washout period, a run-in period of empagliflozin, and then another washout period before being randomized to either empagliflozin or insulin treatment first. After the initial treatment period, participants underwent a third washout periode before receiving the remaining drug (empagliflozin or insulin depending on the randomized order of study drugs)

This was a prospective, open label, two-arm, cross-over study comparing the effects of insulin and empagliflozin treatment titrated to the same glucose level on cardiac function in patients with type 2 diabetes (Fig. 1). The protocol has been described in detail elsewhere [23] and has been publicly available at https://www.clinicaltrialsregister.eu since approval.

Patients were recruited from the Departments of Cardiology and Endocrinology at the Copenhagen University Hospital in Hvidovre, Denmark, and through advertising in local newspapers.

Inclusion criteria were age ≥ 18 years, BMI ≥ 28 kg/m^2^, a diagnosis of diabetes for more than 3 months, HbA1c ≤ 9% (≤ 10% in diet- or metformin-treated only), fasting C-peptide ≥ 500 pmol/L, unchanged glycaemic treatment for 3 months prior to inclusion. The original inclusion criteria of high cardiovascular risk as defined in the EMPA-REG study were waived [1, 23], due to inclusion problems and because later studies reported CVD benefits of SGLT2i treatment in patients with type 2 diabetes with substantially lower cardiovascular risk [24]. Exclusion criteria included prior insulin treatment, renal insufficiency (eGFR < 45 ml/min/1.73 m^2^), persistent atrial fibrillation, claustrophobia, or contraindications to administration of glycopyrrolate. The full list can be found in Additional file 1: Table S1.

Once informed consent was obtained and screening completed, baseline characterization (including echocardiography) was performed. Participants were then taken off all anti-glycemic medications except metformin for an initial 2-week washout period. Other medications were left unchanged. The first washout period was followed by a 2-week empagliflozin run-in period and a second 3-week washout period. Participants were then randomized to treatment with either empagliflozin or NPH insulin first for 5 weeks, followed by a 33-week washout period and the final 5-week treatment period with the remaining study drug. Participants measured fasting glucose during washouts and fasting and pre-prandial evening blood glucose during run-in and treatment periods (Fig. 1).

Empagliflozin was dosed as 25 mg once daily; NPH insulin administered morning and evening was titrated according to target blood glucose (see Additional file 1 for titration algorithm). In participants randomized to insulin first, the glycemic target was average fasting and evening glucose concentrations during the second week of empagliflozin run in. In patients randomized to insulin second, the glycemic target was average fasting and pre-prandial evening blood glucose values during week 3 and 4 of the first (empagliflozin) treatment period.

Participants were studied at the end of the second and third washout periods and at the end of each treatment period for a total of four study visits (V1-4). Each visit consisted of two cardiac MR study days and a metabolic study day.

### Endpoints

The primary endpoint was cardiac diastolic function as determined from left ventricular peak filling rate (LVPFR). Key secondary endpoint was left atrial passive emptying fraction (LAPEF), another measure of cardiac diastolic function. The secondary outcome was left ventricular ejection fraction (LVEF). Exploratory endpoints included change in central blood volume (CBV), left ventricular end-diastolic volume, left ventricular mass and VO2_max_.

We report the between treatments difference (ΔT: empagliflozin—insulin) and the individual treatment effects (ΔE or ΔI: treatment—washout).

### Procedures

Cardiac MR was done at the Dept. of Cardiology, Rigshospitalet, Copenhagen (1.5 T Siemens Aera magnetic resonance scanner). Patients met fasting in the morning, received their morning medication and underwent a cardiac MR scanning protocol, as described previously [23], but without adenosine perfusion scans, due to logistic constraints, and with halving of gadolinium enhancer dose on MR day 1 and omission on MR day 2, due to concerns for CNS accumulation of gadolinium [25].

Surface- and spine coils were used with patients laying supine on their back. Following scout images, cardiac 2 chamber cine images and cardiac short-axis steady-state free precession cine images were obtained. Images were acquired during end-expiratory breath-holds (slice thickness 8 mm (no gap), TE 1.16–1.25 ms, TR 46.24–49.98 ms, matrix 2010–208, FoV 258 × 320–485 × 481, 25 phases). Short-axis images were repeated 10 min after an intravenous bolus injection of the chronotropic stressor glycopyrrolate (4 µg/kg; Robinul^®^, Mylan, Denmark), that has previously been shown to demask diastolic dysfunction [26].

The procedure was repeated on a subsequent study day (MR day 2), where participants were instructed to take 250 mg p.o. acipimox approximately three hours prior to the planned scan to lower FAs by suppressing hormone sensitive lipase activity in adipose tissue [27]. Acipimox administration was repeated before the MR scan to maintain suppression of FAs.

Cardiac diastolic function was determined from the left ventricular peak filling rate (LVPFR) and the left atrial passive emptying fraction (LAPEF). Cardiac systolic function was reported as left ventricular ejection fraction (LVEF). Details on exploratory endpoints are found elsewhere [23] and in the Additional file 1. At each visit, cardiac rhythm was monitored for 48 h (SCOTTCARE, CHROMA, model RZ153C, Cleveland, OH) and ambulatory blood pressure was determined for 24 h (ScottCare, ABP 320, Cleveland, OH).

Metabolic characterization was done by sampling venous blood in the fasting state before and after each cardiac MRI. Additional blood sampling for fasting glucagon measurements, indirect calorimetry (Vyaire, Vyntus^®^ CPX, Chicago, IL, USA) and VO_2, max_ testing (Lode, Corival cpet, Groningen, NL) was performed at the separate metabolic study day [23]. VO_2, max_ testing was modified as described in the Additional file 1.

### Sample collection and biochemical analyses

Arterialized venous blood for analyses of glucose, β-hydroxy butyrate, and Pro Atrial Natriuretic Peptide (ANP)/Brain Natriuretic Peptide (BNP) were collected into pre-chilled EDTA tubes. Blood for analyses of insulin and FA was collected in clot activator tubes. Sampling for aldosterone and renin was done after the participant had rested for 30 min in the supine position. All samples were centrifuged immediately. Plasma glucose was analyzed bedside with the glucose oxidase technique (YSI 2300 STAT Plus, YSI, Yellow Springs, OH, USA). All other samples were centrifuged at 4 °C for 10 min at 4000 rpm, before being aliquoted and frozen at −80 °C. Insulin, FAs and Pro BNP were analyzed on COBAS analyzer **(**Roche Diagostics GmbH, Mannheim, Germany**).** β-hydroxy-butyrate was measured using an enzymatic colorimetric assay (Sigma Aldrich, Merck KGaA, Darmstadt, Germany). Glucagon was measured as previously described [28]. Aldosterone and renin were measured using chemiluminescent immunoassays (iSYS, Immunodiagnostic Systems Holdings Ltd., UK). Pro-ANP was quantified using a processing-independent assay requiring proteolytic treatment prior to measurement [29].

### Statistical analysis

Continuous variables are presented as Mean ± standard error of the mean (SEM) unless otherwise noted. Treatment effects and between treatment differences were tested using the Student´s t-test or Mann–Whitney U test as appropriate. Categorical variables were presented as counts or proportions and compared using Chi-squared test or Fisher’s exact test as appropriate. A two-sided p < 0.05 was considered statistically significant. To test for carry-over effects of treatments, the effects of empagliflozin and insulin on primary and secondary as well as metabolic outcomes were compared between those randomized to insulin and those randomized to empagliflozin first. Statistical analysis was performed with R studio version 1.2.1093 (R Development Core Team). Power calculations have been reported previously [23] and are also available in the Additional file 1.

#### Power calculation

Measures of myocardial function may be determined with a coefficients of variation are in the range of 3–5% using cardiac MRI [30]. Based on data from a previous study using the same Cardiac MRI protocol to determine cardiac function in young elderly healthy subjects [4], we assumed that T2DM patients had LVPFR corresponding to healthy elderly subjects and that empagliflozin treatment would improve LVPFR by 30 ml/s compared to insulin treatment. Estimating SD of between treatment differences of ΔLVPFR at 30 ml/s, a sample size of 16 would be adequate to determine a 30 ml/s difference between the two treatments with a power of 93% and a two-sided significance level of 0.01, when evaluating data with the paired Student’s t-test. We planned to randomize 20 participants, expecting a 20% drop-out rate [23].

## Results

From December 1st 2017 until June 12th 2020, forty-one subjects were screened, twenty-two were randomized and seventeen completed the study (Additional file 1: Figure S1). The final study population (Table 1) was 2/3 male, with an average age of 58 years, a diabetes duration of 8.5 years, a mean HbA1c of 52 mmol/mol, and an average urine albumin/creatinine ratio in the micro-albuminuric range. Seven participants had a history of macrovascular disease. Patients had normal left ventricular systolic and moderately impaired diastolic function as determined from LV peak filling rates. Anti-glycemic and antihypertensive treatments for each participant are listed in Additional file 1: Table S2.

**Table 1:** Patient characteristics at screening, data are Mean ± SEM or median [range]

N	17
Sex (female/male)	4/13
Age (years)	59 [27–74]
BMI (kg/m^2^)	33 ± 0.7
Diabetes duration (years)	9 [2–25]
Blood pressure (mmHg)	144 ± 4/87 ± 2
History of cardiovascular disease (N)	7
HbA1c (mmol/mol)	52 ± 3
Fasting blood glucose (mmol/L)	8.8 ± 0.4
Fasting C-peptide (pmol/L)	1417 ± 107
eGFR (ml/min/1.73 m^2^)	83 ± 3
Urine albumine creatinine ratio (mg/g)	42 ± 19
Pro-BNP (pmol/L)	11 ± 3
E/E’	7.4 ± 2

### Interventions

Participants underwent pre-empagliflozin (PE) washout for 22 ± 2 days with mean fasting finger stick blood glucose concentrations stabilizing at 8.9 ± 0.4 mmol/L for the last 2 weeks. Pre-insulin (PI) washout lasted 21 ± 2 days with mean fasting blood glucose of 8.7 ± 0.3 mmol/L. Empagliflozin (E) and insulin (I) treatment lasted 39 ± 2 and 38 ± 1 days respectively. On empagliflozin treatment, mean fasting and pre-dinner blood glucose decreased to 8.0 ± 0.3 and 8.3 ± 0.3 mmol/L during the final 2 weeks of treatment. Corresponding values on insulin-treatment were 7.9 ± 0.3 and 8.2 ± 0.3 mmol/L. Final insulin dose was 10 ± 1 IU in the morning and 10 ± 1 IU in the evening. Three serious adverse events were recorded in the entire study. All were deemed unrelated to the study intervention (Additional file 1: Table S3).

### Metabolic and anthropometric data

**Fig. 2:**
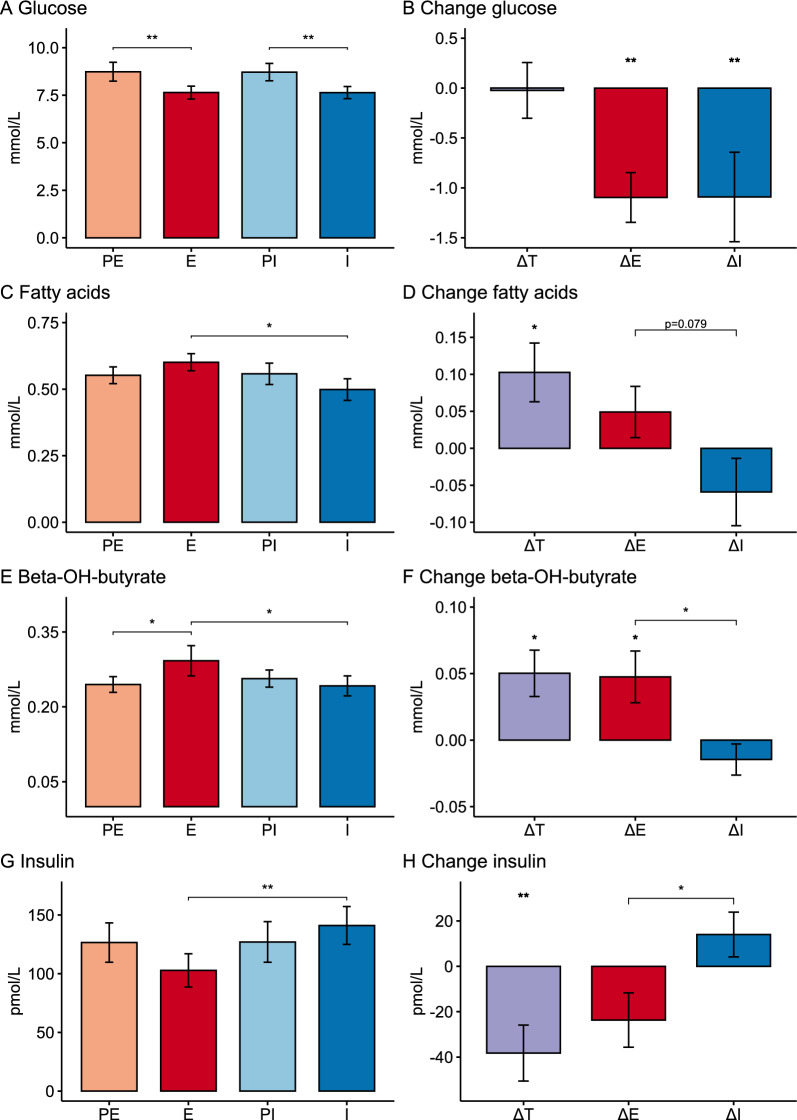
Glucose (panel **A**), fatty acids (panel **C**), β-OH butyrate (panel **E**) and insulin (panel **G**) plasma concentrations before (PE and PI) and during treatments (**E** and **I**). Change in metabolite concentrations (panels **B**, **D**, **F**, **H**) between treatments (ΔT) or between treatment and washout (ΔE and ΔI). Data are Mean ± SEM. *p < 0.05; **p < 0.01

Metabolic data are shown in Table 2 and in Fig. 2. Empagliflozin and insulin treatment reduced fasting glucose similarly compared to washout, from 8.7 mM to 7.6 mM, on the first cardiac MRI day of each treatment visit (Table 2, Fig. 2A, B). Peripheral insulin concentrations decreased on empagliflozin treatment and increased on insulin treatment, resulting in significantly lower insulin concentrations on empagliflozin compared to insulin treatment (Table 2, Fig. 2G, H). FA and β-hydroxybutyrate concentrations on the other hand were higher on empagliflozin than on insulin treatment (Table 2 and Fig. 2B–D). Glucagon concentrations were unchanged at all visits (Table 4), but the glucagon/insulin ratio (PE: 0.19 ± 0.02, E: 0.26 ± 0.04, PI: 0.20 ± 0.03, I: 0.17 ± 0.02) was increased on empagliflozin treatment compared to washout (ΔE: 0.07 ± 0.02, p < 0.01) and insulin treatment (ΔT: 0.09 ± 0.03, p < 0.01). The respiratory quotient was lower during empagliflozin compared with insulin (Table 2) and body weight reduced (ΔE: −1.5 ± 0.4 kg (p = 0.04); ΔI: 1.7 ± 0.3 (p < 0.01), ΔT: −2.2 ± 0.6 kg (p < 0.01)) during empagliflozin treatment compared with washout and insulin.

**Table 2:** Metabolic outcomes

	PE	E	ΔE	PI	I	ΔI	ΔT
MR day 1							
Glucose (mmol/L)	8.7 ± 0.5	7.6 ± 0.3	−1.1 ± 0.2**	8.7 ± 0.5	7.6 ± 0.3	−1.1 ± 0.4*	−0.0 ± 0.3
FAs (mmol/L)	0.55 ± 0.03	0.60 ± 0.03	0.05 ± 0.04	0.56 ± 0.04	0.50 ± 0.05	−0.06 ± 0.05	0.10 ± 0.04*
β-OH butyrate (mmol/L)	0.25 ± 0.02	0.29 ± 0.03	0.05 ± 0.02*	0.26 ± 0.02	0.24 ± 0.02	−0.02 ± 0.01††	0.05 ± 0.02 *
Insulin (pmol/L)	127 ± 17	103 ± 14	−24 ± 11	127 ± 17	141 ± 16	14 ± 10†	−38 ± 12**
Respiratory quotient	0.80 ± 0.01	0.75 ± 0.03	−0.04 ± 0.04	0.84 ± 0.02	0.84 ± 0.02	0.00 ± 0.02	−0.09 ± 0.03*
MR day 2—acipimox							
Glucose (mmol/L)	8.7 ± 0.5	7.6 ± 0.3	−1.1 ± 0.4**	8.1 ± 0.3	7.6 ± 0.3	−0.6 ± 0.2*	−0.1 ± 0.2
FAs (mmol/L)	0.33 ± 0.04‡‡	0.41 ± 0.04‡‡	0.08 ± 0.05	0.34 ± 0.03‡‡	0.32 ± 0.04‡‡	−0.02 ± 0.04	0.10 ± 0.05
Ketone bodies (umol/L)	0.23 ± 0.01	0.25 ± 0.02	0.02 ± 0.01	0.23 ± 0.02‡	0.22 ± 0.02‡	−0.01 ± 0.02	0.04 ± 0.02*
Insulin (pmol/L)	139 ± 24	87 ± 9	−52 ± 19*	106 ± 13	149 ± 23	43 ± 14**††	−62 ± 17**

Data are presented as Mean ± SEM

*PE* Pre-Empagliflozin, *E* Empagliflozin, *PI* Pre-Insulin, *I* Insulin, *ΔE* E-PE, *ΔI* I-PI, *ΔT* E-I

^*^p < 0.05; **p < 0.01; †p < 0.05; ††p < 0.01 vs ΔE. ‡p < 0.05 vs MR Day 1. ‡‡p < 0.01 vs MR Day 1

### Cardiac diastolic and systolic function

During rest, empagliflozin treatment resulted in lower LVPFR but not a lower LAPEF directly compared to insulin, but neither empagliflozin nor insulin treatment changed cardiac diastolic function as measured by LVPFR or LAPEF in the resting state as compared to washout (Table 3). Chronotropic stress eliminated any difference in LVPFR and LAPEF between treatments. Cardiac systolic function (LVEF) did not differ significantly between treatments at any visits during rest or stress (Table 3).

**Table 3:** Cardiac outcomes

	PE	E	ΔE	PI	I	ΔI	ΔT
Cardiac functionṣ—rest							
MR day 1							
LVPFR (ml/s)	400 ± 27	376 ± 28	−38 ± 20	405 ± 33	417 ± 25	8 ± 25	−55 ± 19**
LAPEF (%)	24 ± 2	27 ± 3	2 ± 3	24 ± 3	26 ± 3	1 ± 2	2 ± 2
LVEF (%)	61 ± 2	63 ± 3	2 ± 1	62 ± 2	63 ± 2	1 ± 1	−1 ± 2
Heart Rate (min^−1^)	63 ± 2	63 ± 2	−1 ± 1	65 ± 3	64 ± 2	−1 ± 1	0 ± 1
MR day 2*—*acipimox							
LVPFR (ml/s)	427 ± 25	367 ± 24	−56 ± 30	391 ± 35	386 ± 21	−5 ± 27	−15 ± 25
LAPEF (%)	24 ± 2	26 ± 2	2 ± 2	22 ± 2	26 ± 3	4 ± 2	−0 ± 2
LVEF (%)	59 ± 2	56 ± 2‡‡	−2 ± 2	56 ± 2‡‡	57 ± 2‡‡	1 ± 1	−1 ± 2
Heart Rate (min^−1^)	62 ± 2	64 ± 2	2 ± 2	64 ± 2	61 ± 2‡‡	−2 ± 1*,†	3 ± 1*
Cardiac function–chronotropic stress							
MR day 1							
LVPFR (ml/s)	334 ± 30	334 ± 31	−4 ± 44	367 ± 32	361 ± 30	−16 ± 28	−12 ± 47
LAPEF (%)	20 ± 2	21 ± 3	1 ± 3	21 ± 3	23 ± 3	2 ± 2	−1 ± 1
LVEF (%)	58 ± 2	59 ± 3	−1 ± 2	57 ± 3	57 ± 2	−1 ± 1	1 ± 1
Heart Rate (min^−1^)	78 ± 2	78 ± 2	0 ± 2	78 ± 3	80 ± 2	1 ± 1	−1 ± 2
MR day 2*—*acipimox							
LVPFR (ml/s)	340 ± 28	307 ± 12	−33 ± 26	321 ± 27	358 ± 29	37 ± 26†	−51 ± 22*
LAPEF (%)	22 ± 3	23 ± 3	1 ± 2	21 ± 3	24 ± 3	3 ± 1*	−2 ± 3
LVEF (%)	57 ± 2	54 ± 2‡‡	−3 ± 1*	56 ± 2	57 ± 2	1 ± 2	−3 ± 1*
Heart rate (min^−1^)	78 ± 2	78 ± 2	−1 ± 2	77 ± 3	78 ± 3	1 ± 2	−1 ± 2

Data are presented as Mean ± SEM

*PE* Pre-Empagliflozin, *E* Empagliflozin, *PI* Pre-Insulin, *I* Insulin, *ΔE* E-PE, *ΔI* I-PI, *ΔT* E-I

^*^p < 0.05; **p < 0.01. †p < 0.05 vs ΔE. ‡‡p < 0.01 vs MR Day 1

### Metabolic effects of acipimox

To evaluate the importance of lipid metabolism in cardiac function, cardiac MRI was repeated on a separate day at each visit after acute lowering of plasma FAs for at least 3 h with acipimox. After acipimox administration, glucose and insulin concentrations were unaffected at all visits compared to the first cardiac MRI day, while FAs were reduced about 35% independently of treatments. β-hydroxy butyrate was only numerically reduced (9–12%) during acipimox administration (Table 2).

### Cardiac effects of acipimox

**Fig. 3:**
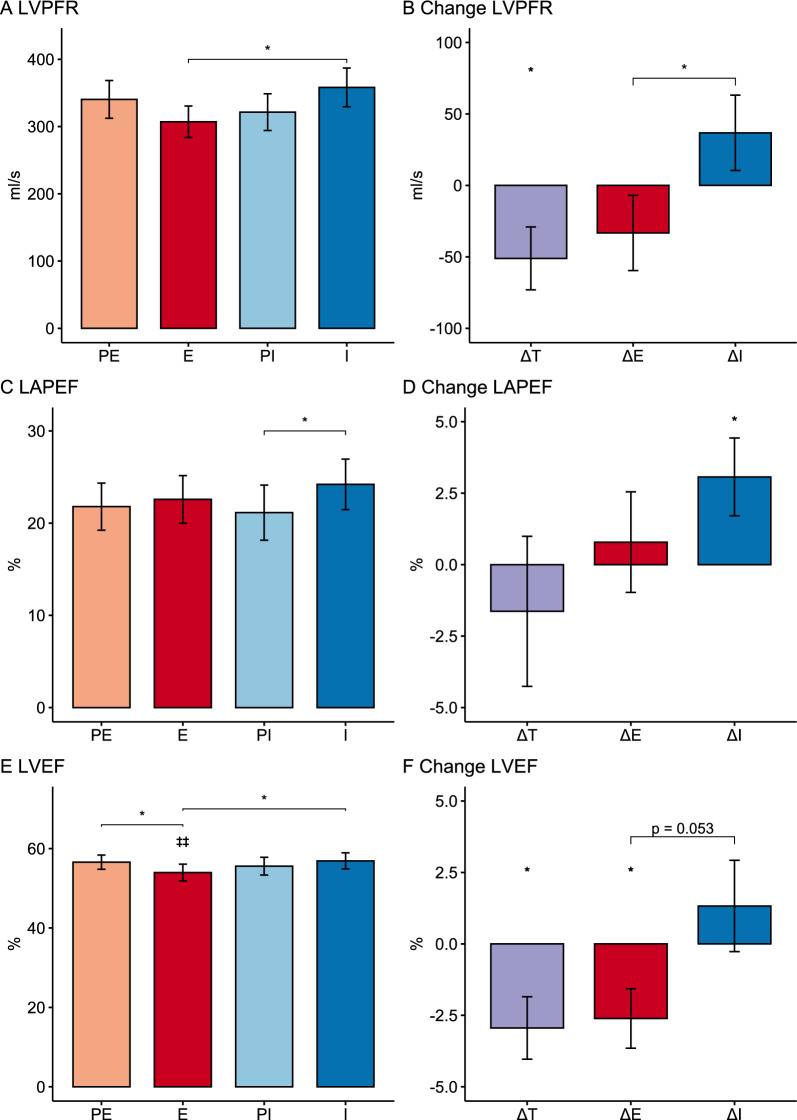
Cardiac diastolic (panel **A**, **C**) and systolic (panel **E**) cardiac function during pharmacologically induced chronotropic stress and acute pharmacological reduction of plasma FAs with acipimox before (PE and PI) and during treatments (**E** and **I**). Change in diastolic (panels **B**, **D**) and systolic (panel **F**) cardiac function between treatments (ΔT) or between treatment and washout (ΔE and ΔI). Data are Mean ± SEM. *p < 0.05. ‡‡p < 0.01 vs MR Day 1. *LVPFR* Left ventricular peak filling rate, *LAPEF* Left atrial passive emptying fraction, *LVEF* Left ventricular emptying fraction

Resting cardiac diastolic function (LVPFR and LAPEF) was unchanged by acute reduction of FAs with acipimox administration, whereas resting LVEF was reduced at all visits compared to the previous cardiac MRI day with higher FAs (Table 3). Chronotropic stress in combination with acipimox resulted in reduced LVPFR on empagliflozin treatment as compared with insulin (Fig. 3A, B). Empagliflozin treatment was associated with numerically impaired and insulin treatment with numerically improved LVPFR compared with washout, and these responses to treatments during chronotropic stress and acipimox administration differed significantly (Fig. 3B). LAPEF was unchanged on empagliflozin treatment but improved on insulin treatment as compared with washout (Fig. 3C, D). During chronotropic stress and acipimox administration LVPFR and LAPEF were unaffected, but LVEF was reduced during empagliflozin treatment as compared to the first MRI day at the same treatment visit with higher FAs (Table 3 and Fig. 3E). Under chronotropic stress and acipimox administration, empagliflozin treatment resulted in lower LVEF than during insulin treatment. Compared with washout (PE) LVEF was significantly impaired during empagliflozin treatment when FAs were pharmacologically reduced and the heart stressed, whereas LVEF numerically increased during insulin treatment compared to washout. These differences in treatment effects were borderline significant (p = 0.05) (Fig. 3E, F).

### Cardiac effects stratified according to initial cardiac function and metabolic response to empagliflozin

To investigate whether baseline cardiac function influenced treatment response, we stratified participants by median into two groups according to cardiac function—one with “good” (top half of population) and one with “poor” (bottom half) cardiac function. We could not demonstrate any systematic differences in the response to treatments or indeed between treatments when stratifying participants according to “good” or “poor” initial diastolic or systolic cardiac function (Additional file 1: Table S4). To evaluate whether the magnitude of the metabolic response to empagliflozin treatment determined any change in cardiac function, we also stratified participants according to the glycemic improvement, reduction in insulin, increase in FA or β-OH butyrate concentrations on empagliflozin treatment, but we could not demonstrate any difference in cardiac functional response between “good” and “poor” metabolic responders (Additional file 1: Table S5).

### Exploratory cardiac endpoints

Central blood volume, left ventricular end-diastolic volume and left ventricular myocardial mass were not significantly changed by 5 weeks of empagliflozin or insulin treatment (Table 4). Cardiac output during chronotropic stress was slightly reduced on empagliflozin compared with insulin treatment (Table 4), and this finding was consistent on MR day 2 with acipimox administration (Additional file 1: Table S6). Ambulatory 24 h blood pressure, pulse-pressure product and VO_2 max_ were essentially similar during washout and treatment periods. While aldosterone, renin and pro-BNP were unchanged throughout the study (Additional file 1: Table S5), we did see a reduction in pro-ANP with empagliflozin treatment compared with washout and insulin treatment (Table 4 and Additional file 1: Table S6). Treatment effects differed on both MR days (ΔE v ΔI: MR1 p = 0.056, MR2 p = 0.029). Holter monitoring did not demonstrate any differences in standard measures of cardiac rhythm across visits (Additional file 1: Table S7).

**Table 4:** Exploratory outcomes

	PE	E	ΔE	PI	I	ΔI	ΔT
MR day 1							
Central blood volume (L)	1077 ± 58	1026 ± 74	−45 ± 67	1132 ± 67	1094 ± 57	−29 ± 43	−61 ± 60
Left ventricular end-diastolic volume (mL)	163 ± 10	160 ± 11	−4 ± 6	166 ± 10	164 ± 10	−3 ± 3	−4 ± 5
Cardiac output Stress (L/min)	6.52 ± 0.38	6.23 ± 0.29	−0.48 ± 0.24	6.24 ± 0.33	6.68 ± 0.29	0.30 ± 0.21	−0.51 ± 0.23*
LV myocardial mass (g/m^2^)	65 ± 4	66 ± 4	0 ± 2	65 ± 4	65 ± 4	0 ± 2	0 ± 1
Pro-ANP (pmol/L)	405 ± 67	340 ± 54	−65 ± 26*	419 ± 65	427 ± 68	8 ± 23	−86 ± 27**
24-h ambulatory blood pressure monitoring							
Systolic BP (mmHg)	130.2 ± 2.8	130.1 ± 1.5	0.9 ± 2.7	130.9 ± 3.6	134.9 ± 3.1	3.9 ± 3.4	−4.7 ± 3.2
Diastolic BP (mmHg)	76.9 ± 1.8	77.41 ± 1.7	1.8 ± 1.4	78.4 ± 2.5	79.2 ± 1.9	1.0 ± 2.3	−0.9 ± 1.6
24-h pulse pressure product (mmHg x min^−1^)	9679 ± 341	10,032 ± 354	402 ± 235	9810 ± 414	10149 ± 433	482 ± 277	−223 ± 305
Metabolic study day							
VO_2_max (ml/kg)	14 ± 0.7	14 ± 0.7	0.1 ± 0.5	16 ± 1	14 ± 0.7	−1.7 ± 0.4†	0.0 ± 0.7
Glucagon (pmol/L)	18 ± 1	19 ± 1	0 ± 1	19 ± 1	19 ± 1	1 ± 1	0 ± 1

Data are presented as Mean ± SEM

*PE* Pre-Empagliflozin, *E* Empagliflozin, *PI* Pre-Insulin, *I* Insulin, *ΔE* E-PE, *ΔI* I-PI, *ΔT* E-I

^*^p < 0.05; **p < 0.01. †p < 0.05 vs ΔE

## Discussion

Here we show that 5 weeks treatment with empagliflozin and insulin titrated to the same level of glycemic control has opposite effects in terms of insulin concentrations and concentrations of FAs and ketone bodies. Thus, compared to insulin treatment, insulin concentrations were ~ 25% lower and FA and β-OH-butyrate concentrations ~ 20% higher on empagliflozin, probably explained by an increased glucagon/insulin ratio on empagliflozin treatment. Based on earlier echocardiographic studies [22], we hypothesized that cardiac diastolic function would improve with 5 weeks of empagliflozin treatment. However, despite clear differences in metabolite concentrations, we found only a slight reduction in LVPFR during rest on empagliflozin treatment compared with insulin. This difference disappeared with chronotropic stress, instead of being accentuated as expected [25], and we therefore conclude that neither diastolic nor systolic cardiac function is consistently changed on empagliflozin treatment compared with insulin treatment. Exploratory data suggest that cardiac metabolism may be switched towards greater reliance on lipid metabolism, which together with a lowered venous return to the heart could contribute to the cardiovascular benefits of SGLT2 inhibitors.

Changes in metabolic parameters of type 2 diabetes may influence cardiac function. For instance, in severely dysregulated patients with type 2 diabetes, 12 months of intensified glycemic control was associated with improved cardiac function, but whether this was simply due to improved glycemia or changes in other parameters of metabolism cannot be determined [31]. The present study was designed to dissect the role of the characteristic metabolic aberrations of type 2 diabetes, hyperglycemia, hyperinsulinemia and hyperlipidemia, for cardiac function. Since cardiac function did not change on either treatment, we conclude that the deleterious effects of mild-to-moderate hyperglycemia, hyperinsulinemia and elevated FAs per se on cardiac function are negligible, at least when present for only 5 weeks.

SGLT2i treatment is associated with hyper-ketonemia, and since ketone bodies are easily converted to energy they may potentially improve cardiac resilience under stressful circumstances [4]. We could not demonstrate any positive effect of empagliflozin, negative effect of insulin, nor difference between the two treatments with respect to cardiac function during chronotropic stress, despite ~ 20% higher β-OH butyrate concentrations during empagliflozin treatment. Infusion of β-OH butyrate improves cardiac contractility in glucose tolerant patients with heart failure, but at the same time plasma levels of β-OH butyrate are several-fold higher than in the present study [21]. The question is whether a physiological increase in ketone body concentrations plays any role for proper cardiac function, at least in patients with normal baseline systolic function as in our cohort.

To further investigate the role of metabolically available lipids for cardiac function, we repeated cardiac functional testing during acute lowering of FAs with acipimox. This resulted in a 35% reduction in FAs and only minor changes in β-OH butyrate concentrations. During chronotropic stress this led to reduced cardiac diastolic and systolic function during empagliflozin treatment compared with insulin treatment, indicating a greater dependence on lipid oxidation for proper cardiac function. At a time when whole body lipid oxidation has increased, as on empagliflozin treatment, it is reasonable to assume that cardiac metabolism also switches towards greater lipid oxidation. A similar dependence on lipid availability for normal cardiac function is seen in normal glucose tolerant subjects [32]. Cardiac phosphocreatine to ATP ratio is reduced in patients with type 2 diabetes at lower blood glucose levels [33]. Increased availability of FAs and lipid oxidation during glucose lowering with empagliflozin treatment, could help counteract this energy depletion and improve outcomes after cardiac ischemia [5]. Whether this has any clinical implications in humans is little investigated, but in the hearts of insulin resistant rats, increasing lipid oxidation improves post-ischemic recovery and vice versa, posing an interesting coupling of metabolic and clinical effects of SGLT2 inhibition [34, 35].

Of interest, pro-ANP concentrations decreased with empagliflozin treatment. This has been shown before [36], and may reflect reduced distention of the atria as a result of a lowered venous return to the heart. Supporting this idea, cardiac output during empagliflozin treatment was lower during chronotropic stress. However, although numerically reduced compared to washout and insulin treatment, we could not demonstrate any significant changes in central blood volume during empagliflozin treatment, and likewise we did not see any change in left ventricular end-diastolic volume. Pro-ANP harbors the hormonally active ANP that stimulates lipolysis [37], but a reverse relation where elevated FAs could inhibit pro-ANP release does not seem to be present, since pro-ANP levels were unaffected by acipimox administration. Hyperinsulinemia and hyperglycemia did not influence pro-ANP release in the present study, but in healthy athletes pro-ANP concentrations decrease during exogenous ketosis [38]. Thus, in combination with reductions in circulating volume and a reduced venous return to the heart, hyperketonemia could potentially contribute to the lower levels of pro-ANP during empagliflozin treatment.

Some studies with longer treatment durations have shown improved diastolic function with SGLT2 inhibitor treatment in patients with T2D, possibly due to reduced left ventricular myocardial mass, whereas others have not [39–42]. The aim of this study was to test how the metabolic effects of SGLT2 inhibition influenced cardiac function independently of structural changes in the heart of patients with T2D. The shorter treatment duration was chosen to reduce the risk of developing cardiac structural changes, while at the same time ensuring robust metabolic differences [18]. The fact that we observed clear metabolic changes without any change in LV myocardial mass indicates that our results can be ascribed to metabolic rather than structural changes in the myocardium. Nevertheless, our findings are in agreement with a recently published study, which also used MRI to evaluate cardiac function in patients randomized to either placebo or dapagliflozin for 6 weeks [43]. In that study, no effect on parameters of diastolic or systolic function nor on left ventricular myocardial mass were observed either.

A major strength of the present study is the use of cardiac MRI, as this is superior to echocardiography in terms of reproducibility and accuracy, and therefore considered the non-invasive reference standard for evaluation of cardiac function [44]. Another strength is the use of insulin as comparator. This results in greater differences with respect to insulin, FA and ketone body concentrations, while at the same time eliminating differences in glycemia as a contributing factor. The use of the cross-over study design provides a high statistical efficiency but does introduce the risk of carry-over effects. Testing for carry-over effects, however, was negative with respect to both cardiac and metabolic outcomes, reflecting adequate washouts between treatment periods.

A limitation of the study is sample size. We planned to include 20 patients, which should enable us to detect a between treatment difference (ΔT) on LVPFR of 30 ml/s. We based our assumptions on effect sizes and data variation on a previous study comparing cardiac MRI measures in healthy young and old people [26]. As it turned out, our population was more heterogenous and with a wider age span probably explaining the greater data variation. Never-the-less at the current number of participants and standard deviations, we still had 80% power to detect 56 ml/s difference in the primary outcome between treatments with a two-sided p-value of < 0.05.

It may be argued that participants did not have “sufficiently impaired” cardiac function to show any effects of SGLT2i. While the clinical benefits of SGLT2i are more pronounced in heart failure, this condition is often associated with substantial remodeling and fibrosis of the left ventricle, which could impair the ability to swiftly improve cardiac function in response to metabolic changes [45–47]. Our primary aim was to demonstrate change in diastolic cardiac function, the earliest measure of diabetic cardiomyopathy. Indeed, our cohort had an E/E’ of7.4 and thus diastolic function was bordering on impaired by standard echocardiographic definitions. However, when considering LV peak filling rates, our participants had lower (~ 25%) values than reported for the normal population in a recent meta-analysis [48], leaving room for treatments to improve cardiac diastolic function. Treatment effects were independent of baseline cardiac function, supporting our conclusions.

Finally, while the design is appropriate for evaluating early metabolic effects of SGLT2 inhibition on cardiac function, it does not allow us to conclude anything regarding effect of long-term SGLT2i treatment.

## Conclusions

We found no evidence of direct effects of SGLT2i induced metabolic changes on cardiac function in patients with type 2 diabetes and moderately impaired diastolic function, when compared to insulin treatment. Impaired diastolic and systolic function during empagliflozin treatment and acute lowering of FAs could suggest greater cardiac reliance on lipid oxidation, which in addition to a lowered venous return to the heart may contribute to the early cardiac benefits of SGLT2 inhibition.

### Supplementary Information

**Additional file 1: Table S1.** Full list of exclusion criteria. Since adenosine perfusion scans were removed from the protocol, exclusion criteria related to that specific procedure were omitted. **Figure S1.** Flow-chart showing patient flow in the study. **Table S2.** Antiglycemic and antihypertensive treatment upon screening in each participant. **Table S3.** Adverse events. Data are number of events while patients were on either treatment or during washout. The number of participants exposed to empagliflozin treatment and to washout is higher than the number of participants exposed to insulin treatment due to the 2-week run-in period and subsequent washout period, that a substantial number of screened patients underwent without entering the study. **Table S4.** Change in cardiac function when stratified into “good” (above) or “poor” (below median value) cardiac function during chronotropic stress at first baseline visit on MR Day 1 (washout visit 1). S3A stratified according to LVPFR. S3B stratified according to LVEF. Data are presented as mean ± sem. ΔE: Empagliflozin—pre-empagliflozin washout; ΔI: Insulin – pre-insulin wahout; ΔT: Empagliflozin-insulin. *: p < 0.05; **: p < 0.01. †: p < 0.05 vs ΔE. **Table S5.** Change in cardiac function when stratified into good or poor glycemic (A), insulin (B), FFA (C) or β-OH butyrate (D) response to empagliflozin treatment. Data are presented as mean ± sem. ΔE: Empagliflozin—pre-empagliflozin washout; ΔI: Insulin – pre-insulin wahout; ΔT: Empagliflozin—insulin. *: p < 0.05; **: p < 0.01. †: p < 0.05 vs ΔE. **Table S6.** Additional exploratory outcomes related to cardiovascular function. Data are presented as mean ± sem. PE: Pre-Empagliflozin; E: Empagliflozin; PI: Pre-Insulin; I: Insulin. ΔE: E-PE; ΔI: I-PI; ΔT: E-I. *: p < 0.05; **: p < 0.01. †: p < 0.05 vs ΔE. **Table S7.** Electrocardiographic data from Holter monitoring. Data are presented as mean ± sem. PE: Pre-Empagliflozin; E: Empagliflozin; PI: Pre-Insulin; I: Insulin. ΔE: E-PE; ΔI: I-PI; ΔT: E-I. *: p < 0.05; **: p < 0.01. †: p < 0.05 vs ΔE.

## Data Availability

The datasets generated and/or analysed during the current study are available in the EudraCT repository: https://eudract.ema.europa.eu/.

## Abbreviations

FA: Fatty acids
SGLT2i: Sodium glucose linked co-transporter 2 inhibitor
LVPFR: Left ventricular peak filling rate
LVEF: Left ventricular ejection fraction
LAPEF: Left atrial passive emptying fraction
LVEDV: Left ventricular end diastolic volume
E: Empagliflozin
I: Insulin
WO: Washout
PE: Pre-empagliflozin treatment washout
PI: Pre-insulin treatment washout
CBV: Central blood volume
MRI: Magnetic resonance imaging
Pro-ANP: Pro atrial natriuretic peptide
