# Supplementary material for: The role of empagliflozin-induced metabolic changes for cardiac function in patients with type 2 diabetes. A randomized cross-over magnetic resonance imaging study with insulin as comparator
Source: Cardiovasc Diabetol. 2024 Jan 6;23:13. doi: 10.1186/s12933-023-02094-x (PMC10771642; doi:10.1186/s12933-023-02094-x)
Supplement: Supplementary file 1 — Additional file 1: Table S1. Full list of exclusion criteria. Since adenosine perfusion scans were removed from the protocol, exclusion criteria related to that specific procedure were omitted. Figure S1. Flow-chart showing patient flow in the study. Table S2. Antiglycemic and antihypertensive treatment upon screening in each participant. Table S3. Adverse events. Data are number of events while patients were on either treatment or during washout. The number of participants exposed to empagliflozin treatment and to washout is higher than the number of participants exposed to insulin treatment due to the 2-week run-in period and subsequent washout period, that a substantial number of screened patients underwent without entering the study. Table S4. Change in cardiac function when stratified into “good” (above) or “poor” (below median value) cardiac function during chronotropic stress at first baseline visit on MR Day 1 (washout visit 1). S3A stratified according to LVPFR. S3B stratified according to LVEF. Data are presented as mean ± sem. ΔE: Empagliflozin—pre-empagliflozin washout; ΔI: Insulin – pre-insulin wahout; ΔT: Empagliflozin-insulin. *: p < 0.05; **: p < 0.01. †: p < 0.05 vs ΔE. Table S5. Change in cardiac function when stratified into good or poor glycemic (A), insulin (B), FFA (C) or β-OH butyrate (D) response to empagliflozin treatment. Data are presented as mean ± sem. ΔE: Empagliflozin—pre-empagliflozin washout; ΔI: Insulin – pre-insulin wahout; ΔT: Empagliflozin—insulin. *: p < 0.05; **: p < 0.01. †: p < 0.05 vs ΔE. Table S6. Additional exploratory outcomes related to cardiovascular function. Data are presented as mean ± sem. PE: Pre-Empagliflozin; E: Empagliflozin; PI: Pre-Insulin; I: Insulin. ΔE: E-PE; ΔI: I-PI; ΔT: E-I. *: p < 0.05; **: p < 0.01. †: p < 0.05 vs ΔE. Table S7. Electrocardiographic data from Holter monitoring. Data are presented as mean ± sem. PE: Pre-Empagliflozin; E: Empagliflozin; PI: Pre-Insulin; I: Insulin. ΔE: E-PE; ΔI: I-PI; ΔT: [file 12933_2023_2094_MOESM1_ESM.docx]

The role of empagliflozin-induced metabolic changes for cardiac function in patients with type 2 diabetes. A randomized cross-over magnetic resonance imaging study with insulin as comparator

Additional file:

RESEARCH DESIGN AND METHODS:

| EXCLUSION CRITERIA:   - Insulin treatment within 3 months of informed consent - Type 1 diabetes - Psychiatric disorder or mental retardation - Drug or alcohol abuse within 3 months from informed consent - Poor compliance - Anemia (hgb ≤ 6.4 mmol/L) or other blood dyscrasias causing hemolysis or unstable red blood cells - Indication of liver disease, defined by serum levels of alanine amininotransferase, aspartate aminotransferase, or alkaline pho, sphatase above 3 x upper limit - Impaired renal function (eGFR<45ml/min/1.73 m2) - Treatment with anti-obesity drugs 3 months prior to informed consent - Systemic steroid treatment within 6 weeks of informed consent - Uncontrolled thyroid disease (prescribed changes in thyroid medication within the 6 weeks prior to informed consent) - Any uncontrolled endocrine disorder except type 2 diabetes - Bariatric surgery or other gastrointestinal disorders that compromises gastrointestinal absorption - Peptic ulcer – verified by endoscopically - Any form of planned surgery within 3 months of informed consent - Acute coronary syndrome, stroke or TCI within 2 months prior to informed consent - Persistent atrial fibrillation - Inability to undergo experimental procedures including exclusion criteria for MRI scanning: - Implantable cardioverter defibrillator/pacemaker - Ferromagnetic clips - Claustrophobia. - Contraindication to glycopyrrolate infusion: - Known closed-angle glaucoma - known severe prostate hyperplasia - Tachycardia (HR > 100 at rest) - Known bladder atony - Cardia insufficiency or non-congenital pylorus stenosis – verified endoscopically - Known gastroparesis - Allergy towards any of the drugs or diagnostics used in the protocol (insulin, empagliflozin, acipimox, glycopyrrolate and~~, adenosine,~~ gadolinium contrast enhancer). - Any condition which in the opinion of the investigator may jeopardize subject safety or compliance with the protocol |
| --- |

Table S1. Full list of exclusion criteria. Since adenosine perfusion scans were removed from the protocol, exclusion criteria related to that specific procedure were omitted.

*Insulin titration algorithm:*

NPH insulin was started at a dose of 0.2 IU/kg body weight/day and was titrated daily over phone (phone contacts; Figure 1) by 0.05 IU/kg body weight/day until average blood glucose over three consecutive days was within ±1 mmol/L of the individual glycemic target. Average fasting and pre-dinner evening glucose concentrations during the second week of empagliflozin run-in served as glycemic target in the patients randomized to insulin first. Average fasting and pre-dinner evening blood glucose values of weeks three and four of the first study drug period served as glycemic target for insulin treatment in those randomized to empagliflozin first. A numeric difference between median glucose on empagliflozin and insulin treatments of >1.5 mmol/L, was pre-specified as the level at which glycemic control differed and participants were excluded from the study.

*Procedure for measurement of VO2 max:*

We originally planned to estimate maximum oxygen uptake (VO_2_max) at screening and during the study using Aastrands two-point test. However, because of repeated test failures in the first 6 participants, who could not complete an initial 30 min of ergometer cycling exercise at 50% of VO2 max, planned prior to VO2max testing, due to exhaustion, VO2max was instead estimated as oxygen consumption per kilogram body weight during the final minute before the end of exercise in these patients.

In the remainder of the included participants, VO2max was assessed by maximal exercise test. Participants were exercised at 30 W for 3 minutes after which workload was increased with 15 W per minute until two out three conditions were met: RER of 1.15 was reached, the patient no longer could keep up with the rotational speed (60-80 rotation/minute) or a VO_2_ plateau was obtained despite an increased muscular work (1). Heart rate, VO_2_-uptake and indirect calorimetric values were registered and saved during the entire test.

*Cardiac MR study days:*

In post-processing analysis (third-part software cvi42, Circle cardiovascular imaging, Calgary Canada, v.5.13.5) LA and LV time-volume curves were generated from the shot-axis cine images by semi-automatic tracing of the endocardial borders in all 25 phases. Maximal (LAmax), minimal (LAmin) and mid-diastolic LA volumes (LAmdv) were determined. The LA total emptying fraction (LAEF) was calculated as (LAmax – LAmin)/LAmax * 100%, and the LA passive emptying fraction (LAPEF) was determined as (LAmax – LAmdv)/LAmax * 100%. The LV ePFR was generated automatically from the LV time-volume curves. The LV mass was determined from the myocardial density (1.06 g/mL) and the myocardial volume was generated by drawing epicardial contours. Volumes and LV myocardial mass were indexed to body surface area (BSA; Mosteller). T1-mapping was performed at a basal and mid-ventricular short-axis slice using a shortened modified Look-Locker inversion recovery sequence.

Post-processing analyses: The pulmonary blood volume was calculated by multiplying the cardiac output (determined from LVSV multiplicated by the heart rate) and the pulmonary transit time (pulmonary blood volume = CO (mL s−1) x PTT (sec). Pulmonary transit times were determined from the perfusion scans from the pulmonary transit times for gadolinium. Circular regions of interest (ROIs) were drawn over the blood pool of the RV and LV, respectively, corresponding to the mid-ventricular slice. Time-intensity curves were generated for the transit of the gadolinium bolus through the RV and LV ROIs. The RV to LV transit time used to determine PTT was calculated by subtracting the time points when signal intensity in the RV and LV ROIs reached 40% of their respective maxima. Assuming insignificant extravasation of gadolinium during the first pulmonary pass of the gadolinium bolus, we were able to apply indicator dilution theory as previously documented during breath-hold diving (2). Central blood volume was determined as the sum of the pulmonary blood volume and the sum of all heart chambers in ventricular diastole (3)

RESULTS

41 candidates screened

22 particiants randomized

19 fulfilled exclusion criteria

5 participants dropped out

17 particiants completed the study

Figure S1: Flow-chart showing patient flow in the study.

|  | **Antiglycemic treatment** | | | | | **Antihypertensive treatment** | | | | **Antihyperlipidemics** |
| --- | --- | --- | --- | --- | --- | --- | --- | --- | --- | --- |
| **Pt no.** | **MET** | **SU** | **DPP4i** | **SGLT2i** | **GLP-1RA** | **ACEi/AT2A** | **Beta-B** | **CaCi** | **Thiazide** | **Statins** |
| **1** | **X** |  |  |  | **X** | **X** | **X** | **X** |  | **X** |
| **2** | **X** |  |  | **X** | **X** | **X** |  | **X** |  | **X** |
| **3** | **X** |  |  |  |  | **X** | **X** |  |  |  |
| **4** | **X** |  |  |  |  |  |  |  |  |  |
| **5** | **X** |  | **X** |  |  |  |  |  |  | **X** |
| **6** | **X** |  |  |  |  |  |  |  |  |  |
| **7** | **X** |  |  | **X** | **X** | **X** | **X** | **X** |  | **X** |
| **8** | **X** |  |  |  | **X** | **X** | **X** | **X** |  | **X** |
| **9** | **X** |  |  |  |  | **X** |  |  |  | **X** |
| **10** | **X** |  |  | **X** | **X** |  |  |  |  | **X** |
| **11** |  |  |  | **X** | **X** | **X** |  |  |  |  |
| **12** | **X** | **X** |  |  |  | **X** |  |  |  | **X** |
| **13** | **X** |  |  |  |  |  |  |  |  | **X** |
| **14** |  |  | **X** | **X** |  | **X** |  |  |  | **X** |
| **15** | **X** |  | **X** |  |  | **X** | **X** | **X** |  | **X** |
| **16** | **X** |  |  | **X** |  | **X** |  |  |  | **X** |
| **17** | **X** |  |  |  |  |  |  | **X** | **X** | **X** |

Table S2: Antiglycemic and antihypertensive treatment upon screening in each participant.

|  | Empagliflozin (n=29) | Insulin (n=17) | Washout (n=29) |
| --- | --- | --- | --- |
| Participants with ≥ 1 treatment emergent adverse event | 4 | 1 | 10 |
| Serious adverse event | 1 | 0 | 2 |
| Death | 0 | 0 | 0 |
| AEs leading to discontinuation | 0 | 0 | 1 |
| Genital mycotic infection | 2 | 0 | 0 |
| Fatigue | 0 | 0 | 2 |
| Bleeding/pain related to biopsy | 0 | 1 | 2 |
| Allergy | 0 | 0 | 3 |
| RBBB | 1 | 0 | 0 |
| Chest pain | 0 | 0 | 1 |
| Low hemoglobin | 0 | 0 | 1 |
| Psychiatric illness | 1 | 0 | 0 |

Table S3: Adverse events. Data are number of events while patients were on either treatment or during washout. The number of participants exposed to empagliflozin treatment and to washout is higher than the number of participants exposed to insulin treatment due to the 2-week run-in period and subsequent washout period, that a substantial number of screened patients underwent without entering the study.

Three serious adverse events were recorded during the study, an allergic reaction, chest pain and psychiatric illness. Two out of three serious adverse events (SAE) happened during washout, while one SAE happened during empagliflozin treatment (psychiatric illness) – all SAEs were unrelated to treatments. One SAE (allergic reaction)led to withdrawal of the participant from the study, while the two other SAEs had no consequences.

| Table S4A | Δ Empagliflozin | Δ Insulin | Δ Treatments (Empa-Ins) |
| --- | --- | --- | --- |
| **Rest** |  | | |
|  | Good diastolic function ≥ 306 ml/s (stress) | | |
| LVPFR (ml/s) | -53 ± 29 | 16 ± 26 | -67 ± 35 |
| LAPEF (%) | 1 ± 5 | 1 ± 2 | 3 ± 4 |
| LVEF (%) | 2 ± 1 | 0 ± 2 | 2 ± 2 |
|  | Poor diastolic function < 306 ml/s (stress) | | |
| LVPFR (ml/s) | -25 ± 28 | 0 ± 44 | -45 ± 21 |
| LAPEF (%) | 4 ± 4 | 1 ± 5 | 2 ± 3 |
| LVEF (%) | 2 ± 1 | 1 ± 2 | -3 ± 2 |
| **Stress** |  | | |
|  | Good diastolic function LVPFR ≥ 306 ml/s (stress) | | |
| LVPFR (ml/s) | -60 ± 73 | -37 ± 38 | -56 ± 73 |
| LAPEF (%) | 3 ± 4 | 3 ± 3 | -1 ± 2 |
| LVEF (%) | -2 ± 3 | -2 ± 1 | 2 ± 3 |
|  | Poor diastolic function LVPFR < 306 ml/s (stress) | | |
| LVPFR (ml/s) | 44 ± 54 | 2 ± 42 | 28 ± 63 |
| LAPEF (%) | 0 ± 5 | 0 ± 4 | -1 ± 1 |
| LVEF (%) | 1 ± 2 | 0 ± 1 | 1 ± 2 |

| S4B | Δ Empagliflozin | Δ Insulin | Δ Treatments (Empa-Ins) |
| --- | --- | --- | --- |
| **Rest** |  | | |
|  | Good systolic function ≥ 57% (stress) 1 baseline visit | | |
| LVPFR (ml/s) | -94 **± 7** | -19 ± 25 | -51 ± 25 |
| LAPEF (%) | -1 ± 5 | 2 ± 1 | 2 ± 2 |
| LVEF (%) | 2 ± 2 | 0 ± 2 | 2 ± 2 |
|  | Poor systolic function < 57 % (stress) 1 baseline visit | | |
| LVPFR (ml/s) | **27 ± 24 (p=0.002 vs Good)** | 42 ± 47 | -61 ± 31 |
| LAPEF (%) | 5 ± 4 | 0 ± 5 | 2 ± 4 |
| LVEF (%) | 2 ± 1 | 1 ± 2 | 3 ± 2 |
| **Stress** |  | | |
|  | Good systolic function ≥ 57% (stress) 1 baseline visit | | |
| LVPFR (ml/s) | -37 ± 72 | -28 ± 49 | -28 ± 74 |
| LAPEF (%) | 1 ± 3 | 4 ± 3 | 0 ± 2 |
| LVEF (%) | -2 ± 3 | -1 ± 1 | 3 ± 3 |
|  | Poor systolic function < 57 % (stress) 1 baseline visit | | |
| LVPFR (ml/s) | 33 ± 53 | -2 ± 28 | 7 ± 61 |
| LAPEF (%) | 2 ± 5 | -1 ± 4 | -2 ± 2 |
| LVEF (%) | 0 ± 3 | -1 ± 1 | 0 ± 1 |

Table S4: Change in cardiac function when stratified into “good” (above) or “poor” (below median value) cardiac function during chronotropic stress at first baseline visit on MR Day 1 (washout visit 1). S3A stratified according to LVPFR. S3B stratified according to LVEF. Data are presented as mean ± sem. ΔE: Empagliflozin - pre-empagliflozin washout; ΔI: Insulin – pre-insulin wahout; ΔT: Empagliflozin-insulin. *: p<0.05; **: p<0.01. †: p<0.05 vs ΔE.

| Table S5A | Δ Empagliflozin | Δ Insulin | Δ Treatments (Empa-Ins) |
| --- | --- | --- | --- |
| **Rest** |  | | |
|  | Good glycemic response < -0.66 mM | | |
| LVPFR (ml/s) | -43 ± 29 | 4 ± 44 | -60 ± 20 |
| LAPEF (%) | 2 ± 5 | 1 ± 2 | 3 ± 3 |
| LVEF (%) | 2 ± 1 | 1 ± 2 | 0 ± 2 |
|  | Poor glycemic respons > -0.66 mM | | |
| LVPFR (ml/s) | -30 ± 27 | 12 ± 27 | -49 ± 40 |
| LAPEF (%) | 2 ± 5 | 1 ± 4 | 1 ± 4 |
| LVEF (%) | 2 ± 1 | 1 ± 1 | -1 ± 3 |
| **Stress** |  | | |
|  | Good glycemic response < -0.66 mM | | |
| LVPFR (ml/s) | 26 ± 70 | -28 ± 48 | 41 ± 74 |
| LAPEF (%) | 2 ± 3 | 3 ± 3 | -2 ± 1 |
| LVEF (%) | -3 ± 2 | -2 ± 1 | 2 ± 1 |
|  | Poor glycemic respons > -0.66 mM | | |
| LVPFR (ml/s) | -49 ± 40 | -3 ± 30 | -91 ± 21 |
| LAPEF (%) | 1 ± 5 | 0 ± 4 | 0 ± 3 |
| LVEF (%) | 3 ± 4 | 0 ± 1 | 1 ± 3 |

| S5B | Δ Empagliflozin | Δ Insulin | Δ Treatments (Empa-Ins) |
| --- | --- | --- | --- |
| **Rest** |  | | |
|  | Good insulin response < -20 pM | | |
| LVPFR (ml/s) | -56 ± 21 | -28 ± 23 | -41 ± 23 |
| LAPEF (%) | 7 ± 5 | 2 ± 2 | 5 ± 3 |
| LVEF (%) | 2 ± 1 | 0 ± 1 | 0 ± 2 |
|  | Poor insulin response > -20 pM | | |
| LVPFR (ml/s) | -17 ± 35 | 54 ± 45 | -72 ± 32 |
| LAPEF (%) | -3 ± 4 | 0 ± 4 | -1 ± 4 |
| LVEF (%) | 2 ± 1 | 1 ± 2 | -1 ± 3 |
| **Stress** |  | | |
|  | Good insulin response < -20 pM | | |
| LVPFR (ml/s) | -17 ± 53 | -60 ± 35 | 61 ± 63 |
| LAPEF (%) | -3 ± 4 | 2 ± 4 | -3 ± 1 |
| LVEF (%) | 0 ± 2 | -1 ± 1 | 3 ± 2 |
|  | Poor insulin response > -20 pM | | |
| LVPFR (ml/s) | 11 ± 79 | **49 ± 35*** | -94 ± 61 |
| LAPEF (%) | 5 ± 4 | 1 ± 3 | 1 ± 2 |
| LVEF (%) | -1 ± 4 | 0 ± 1 | 0 ± 1 |

| S5C | Δ Empagliflozin | Δ Insulin | Δ Treatments (Empa-Ins) |
| --- | --- | --- | --- |
| **Rest** |  | | |
|  | Good FFA response > 0.04 mM | | |
| LVPFR (ml/s) | -33 ± 27 | 29 ± 40 | -52 ± 28 |
| LAPEF (%) | 2 ± 4 | -2 ± 3 | 2 ± 4 |
| LVEF (%) | 4 ± 1 | 2 ± 2 | -2 ± 3 |
|  | Poor FFA response < 0.04 mM | | |
| LVPFR (ml/s) | -43 ± 31 | - 19 ± 24 | -59 ± 29 |
| LAPEF (%) | 3 ± 5 | 6 ± 3 | 2 ± 2 |
| LVEF (%) | **0 ± 1*** | -1 ± 2 | 1 ± 1 |
| **Stress** |  | | |
|  | Good FFA response > 0.04 mM | | |
| LVPFR (ml/s) | 26 ± 48 | -21 ± 34 | 30 ± 62 |
| LAPEF (%) | 0 ± 3 | 4 ± 2 | -2 ± 2 |
| LVEF (%) | 2 ± 3 | -1 ± 1 | 3 ± 2 |
|  | Poor FFA response < 0.04 mM | | |
| LVPFR (ml/s) | -38 ± 82 | -9 ± 53 | -59 ± 74 |
| LAPEF (%) | **3 ± 5*** | -2 ± 5 | 1 ± 2 |
| LVEF (%) | -4 ± 2 (p=0.09) | 0 ± 1 | -1 ± 2 |

| S5D | Δ Empagliflozin | Δ Insulin | Δ Treatments (Empa-Ins) |
| --- | --- | --- | --- |
| **Rest** |  | | |
|  | Good beta-OH butyrate response > 0.02 mM | | |
| LVPFR (ml/s) | -71 ± 14 | 4 ± 27 | -81 ± 32 |
| LAPEF (%) | 3 ± 6 | 0 ± 4 | 2 ± 4 |
| LVEF (%) | 2 ± 1 | 0 ± 1 | 0 ± 2 |
|  | Poor beta-OH butyrate response < 0.2 mM | | |
| LVPFR (ml/s) | 1 ± 35 | 13 ± 48 | -26 ± 15 |
| LAPEF (%) | 1 ± 3 | 3 ± 2 | 2 ± 3 |
| LVEF (%) | 2 ± 1 | 2 ± 2 | -1 ± 3 |
| **Stress** |  | | |
|  | Good beta-OH butyrate response > 0.02 mM | | |
| LVPFR (ml/s) | -90 ± 66 | -13 ± 39 | -85 ± 65 |
| LAPEF (%) | -5 ± 4 | -3 ± 3 | -4 ± 2 |
| LVEF (%) | 2 ± 3 | -1 ± 1 | 2 ± 2 |
|  | Poor beta-OH butyrate response < 0.2 mM | | |
| LVPFR (ml/s) | 94 ± 36* | -20 ± 44 | 73 ± 58 |
| LAPEF (%) | 7 ± 3* | 7 ± 2 | 1 ± 2* |
| LVEF (%) | -4 ± 2* | 0 ± 1 | 0 ± 1 |

Table S5. Change in cardiac function when stratified into good or poor glycemic (A), insulin (B), FFA (C) or β-OH butyrate (D) response to empagliflozin treatment. Data are presented as mean ± sem. ΔE: Empagliflozin - pre-empagliflozin washout; ΔI: Insulin – pre-insulin wahout; ΔT: Empagliflozin - insulin. *: p<0.05; **: p<0.01. †: p<0.05 vs ΔE.

| Table S6 | PE | E | ΔE | PI | I | ΔI | ΔT |
| --- | --- | --- | --- | --- | --- | --- | --- |
| Cardiac output - Rest MR1 (L/min) | 6.18 ± 0.36 | 6.25 ± 0.41 | -0.10 ± 0.21 | 6.40 ± 0.39 | 6.56 ± 0.38 | -0.03 ± 0.23 | -0.42 ± 0.21 |
| Cardiac output - Rest MR2 (L/min) | 5.93 ± 0.28 | 5.63 ± 0.34 | -0.17 ± 0.19 | 5.75 ± 0.35 | 5.78 ±0.28 | 0.03 ± 0.14 | -0.15 ± 0.17 |
| Cardiac output - Stress MR2 (L/min) | 6.32 ± 0.31 | 5.99 ± 0.27 | -0.34 ± 0.15 * | 6.18 ± 0.30 | 6.33 ± 0.32 | 0.16 ± 0.22 | -0.34 ± 0.16 * |
| LV myocardial mass (g) | 143 ± 9 | 146 ± 10 | 1 ± 5 | 143 ±10 | 145 ± 10 | 2 ± 4 | -2 ± 3 |
| Daytime systolic BP (mmHg) | 130.8±2.8 | 130.6±1.6 | 0.9±2.8 | 130.9±3.7 | 135.5±3.1 | 4.5±3.5 | -4.8±3.2 |
| Daytime diastolic BP (mmHg) | 77.5±1.9 | 78.2±1.8 | 2.0±1.5 | 78.9±2.5 | 79.9±1.9 | 1.2±2.4 | -0.9±1.7 |
| Nighttime systolic BP (mmHg) | 126.9±3.2 | 126.2±2.7 | 0.7±3.1 | 131.2±3.6 | 131.9±4.0 | 1.0±3.6 | -5.5±4.2 |
| Nighttime diastolic BP (mmHg) | 72.8±2.3 | 72.0±1.8 | -0.6±2.0 | 74.5±2.7 | 75±2.2 | 0.5±2.0 | -3.0±2.2 |
| Daytime pulse-pressure product | 9857±349 | 10192±358 | 393±264 | 9927±440 | 10368±449 | 583±313 | -289±360 |
| Nighttime pulse-pressure product | 8853±424 | 9069±470 | 189±411 | 9180±332 | 9102±496 | 32±358 | -166±356 |
| Aldosterone (pmol/L) | 199 ± 30 | 478 ±173 | 273 ± 170 | 192 ± 31 | 246 ± 44 | 63 ± 34 | 223 ± 174 |
| Renin (mIU/L) | 26 ± 4 | 26 ±6 | 0 ± 5 | 32 ± 11 | 36 ± 11 | 4 ± 12 | -11 ± 11 |
| Pro-BNP (pmol/L) | 11 ± 3 | 10 ± 2 | -1 ± 2 | 10 ± 2 | 12 ± 3 | 2 ± 2 | -2 ± 2 |
| Pro-ANP MR2 (pmol/L) | 412 ± 68 | 337 ± 54 | -75 ± 33* | 394 ± 54 | 441 ± 68 | 48 ± 27† | -104 ± 32** |

Table S6. Additional exploratory outcomes related to cardiovascular function. Data are presented as mean ± sem. PE: Pre-Empagliflozin; E: Empagliflozin; PI: Pre-Insulin; I: Insulin. ΔE: E-PE; ΔI: I-PI; ΔT: E-I. *: p<0.05; **: p<0.01. †: p<0.05 vs ΔE.

| Table S7 | PE | E | ΔE | PI | I | ΔI | ΔT |
| --- | --- | --- | --- | --- | --- | --- | --- |
| HR_max_  (min^-1^) | 129± 5 | 122±4 | -7±4 | 118±5 | 120±4 | 2±3 | -11±6 |
| HR_min_  (min^-1^) | 49± 2 | 50±3 | 2±2 | 51±3 | 47±1 | -1±1 | 2±2 |
| HR_average_ (min^-1^) | 78±3 | 75±3 | -1±2 | 78±2 | 76±2 | 0±1 | -3±3 |
| SVES/hour | 16±13 | 10±6 | -7±15 | 5±2.6 | 30±18 | 27±18 | -47±37 |
| VES/hour | 46±24 | 31±17 | -16±16 | 33±19 | 41±21 | 11±15 | -33±39 |
| R-on-T | 21±15 | 1±0 | -24  ±17 | 28±25 | 35±32 | 7±44 | -36  ±63 |

Table S7. Electrocardiographic data from Holter monitoring. Data are presented as mean ± sem. PE: Pre-Empagliflozin; E: Empagliflozin; PI: Pre-Insulin; I: Insulin. ΔE: E-PE; ΔI: I-PI; ΔT: E-I. *: p<0.05; **: p<0.01. †: p<0.05 vs ΔE.

REFERENCES:

1. Beltz NM, Gibson AL, Janot JM, Kravitz L, Mermier CM, Dalleck LC. Graded Exercise Testing Protocols for the Determination of VO 2 max: Historical Perspectives, Progress, and Future Considerations . J Sports Med. 2016;2016:1–12.

2. Mijacika T, Kyhl K, Frestad D, Otto Barak F, Drvis I, Secher NH, et al. Effect of pulmonary hyperinflation on central blood volume: An MRI study. Respir Physiol Neurobiol. 2017;243(June):92–6.

3. by Robert Ginsburg E, Geschwind HJ, Stiikwerda S, Loring Rowell BB. Human cardiovascular control: Edited by Loring B. Rowell, Oxford University Press, New York (1993) ISBN: 9-19-507362-2. Clin Cardiol. 1994 Feb 1

4. Ahtarovski K a, Iversen KK, Lønborg JT, Madsen PL, Engstrøm T, Vejlstrup N. Left atrial and ventricular function during dobutamine and glycopyrrolate stress in healthy young and elderly as evaluated by cardiac magnetic resonance. Am J Physiol Heart Circ Physiol. 2012 Dec 15;303(12):H1469-73.
